# Right coronary artery originated from left coronary sinus associated with children hypertrophic cardiomyopathy: report of two cases and literature review

**DOI:** 10.1515/med-2025-1287

**Published:** 2026-01-19

**Authors:** Meng Xu, Tingting Xiao, Cuilan Hou, Xunwei Jiang, Li Zhang, Lijian Xie

**Affiliations:** Department of Cardiology, Shanghai Children’s Hospital, School of Medicine, Shanghai Jiaotong University, Shanghai, China; Department of Pediatrics, Jinshan Hospital, Fudan University, Shanghai, China

**Keywords:** hypertrophic cardiomyopathy, coronary artery, *TTN*, *MYBPC3*, mutation

## Abstract

**Objectives:**

Coronary artery anomalies are rare both in coronary angiogram and computed tomography angiography. Hypertrophic cardiomyopathy (HCM) is the most frequent inherited cardiac disease. The phenotype of HCM associated with anomalous coronary origin is not commonly seen especially in children.

**Case presentation:**

We describe a case series of two children with HCM combined right coronary artery (RCA) originated from left coronary sinus. Case 1 was a 9-month-old female with *TTN* gene heterozygous mutation (*p.R16724L*) who exhibited cardiac insufficiency. Case 2 was a 12-year-old male with *MYBPC3* gene heterozygous mutation (*p.R820Q*) who only exhibited intermittent chest pain. A total of 7 HCM cases with RCA originated from left coronary sinus have been reported with our literature review. Case 1 is the youngest child patient in our report until now. Moreover, the echocardiogram of case 1 is similar with restrictive cardiomyopathy (RCM) and it demonstrates the progression of HCM to heart failure. So, HCM with *TTN* gene mutation may exhibit cardiac insufficiency more early. And the gene mutation site of *TTN* has never been reported in previous HCM cases.

**Conclusions:**

HCM coexisted with anomalous origin of RCA has different clinical presentation, and it maybe due to different gene mutation.

## Introduction

Coronary artery anomalies (CAA) are rare with 0.76–1.3 % prevalence in coronary angiogram [1], [2], [3]. The incidence of anomalous coronary artery originated from contralateral coronary sinus accounted for 1.7 % in coronary computed tomography angiography (CTA) [4]. Hypertrophic cardiomyopathy (HCM) is the most frequent inherited cardiac disease with a prevalence of approximately 1 in 500 adults. The combination of HCM and anomalous coronary origin is not commonly seen [5], [6], [7], [8], [9], [10], [11]. Whether anomalous coronary origin aggravating the HCM patient’s symptoms is not well known, especially in children. Moreover, the relationship between genotype and phenotype is not clear. Here, we presented 2 child patients with the coexistence of HCM with right coronary artery (RCA) originated from left coronary sinus, both are associated with HCM related gene mutation.

## Case presentation

### Case 1

**Figure 1:**
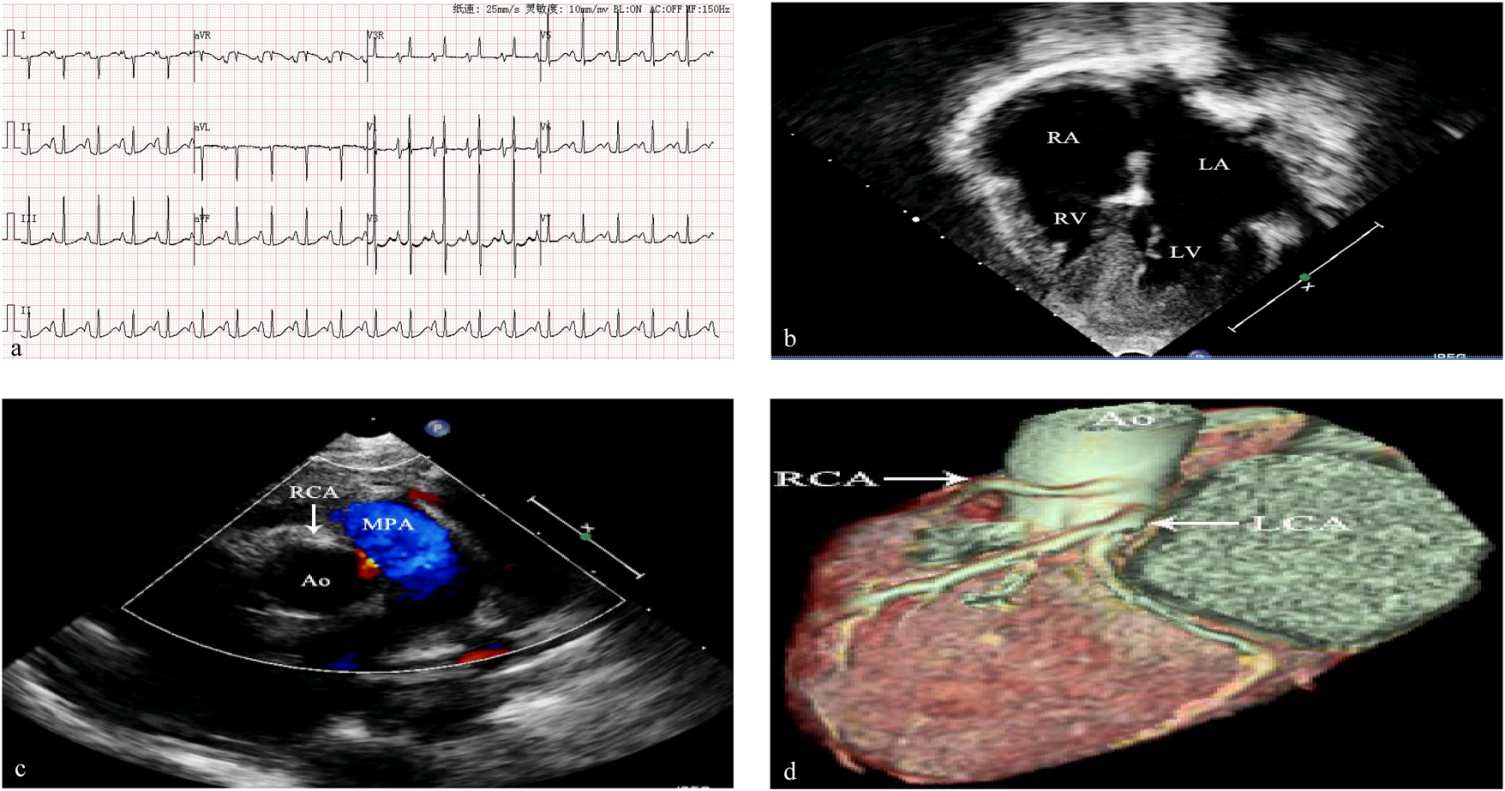
a EKG showed sinus rhythm, double atrial and right ventricular hypertrophy, prolonged PR and QT duration time, and ST segment changes. b Echocardiography depicted the dilatation of the atria, the hypertrophy of ventricular muscle, and two smaller ventricle. c Echocardiography demonstrated the malignant course of the anomalous RCA between the aorta and pulmonary artery. d Computed tomography coronary angiography revealed RCA originated from left coronary sinus.

A 9-month-old female was admitted to our hospital for poor spirits, oliguria, and edema of lower limbs for 10 days after respiratory tract infection. There was no family history of sudden cardiac death, HCM or coronary artery disease. On physical assessment, her respiratory rate was 32 per minute, heart rate was 135 per minute, blood pressure was 89/56 mmHg and SpO_2_ was 99 %. Her body weight was 8 kg. Physical examination of the heart and lungs was unremarkable. Laboratory tests yielded a normal complete blood count, basic metabolic profile, normal troponin levels, however, NT-proBNP increased more than 15,000 pg/mL (Reference range of NT-proBNP is 0∼250 pg/mL). The tandem mass spectrum of blood and urine was normal. An electrocardiogram (ECG) revealed sinus rate, double atrial and right ventricular hypertrophy, prolonged PR (PR interval=174 ms) and QT duration time (QT interval=458 ms), and ST segment changes (Figure 1a). The echocardiogram revealed enlarged left and right atria, double ventricular hypertrophy, interventricular septal thickness was 1.50 cm (z-score=26.38) (http://zscore.chboston.org/) in diastole and 1.55 cm (z-score=13.32) in systole, left ventricular free wall thickness was 0.85 cm (z-score=8.44) in diastole and 0.88 cm (z-score=2.51) in systole, anomalous right coronary artery origin and low eject fraction (EF=44 %) (Figure 1b and c). Coronary CTA revealed RCA was originated from left sinus valsalva and RCA main trunk was stenosis (Figure 1d). The patient was discharged with oral diuretics. She died suddenly 9 months later while waiting for heart transplantation.

### Case 2

**Figure 2:**
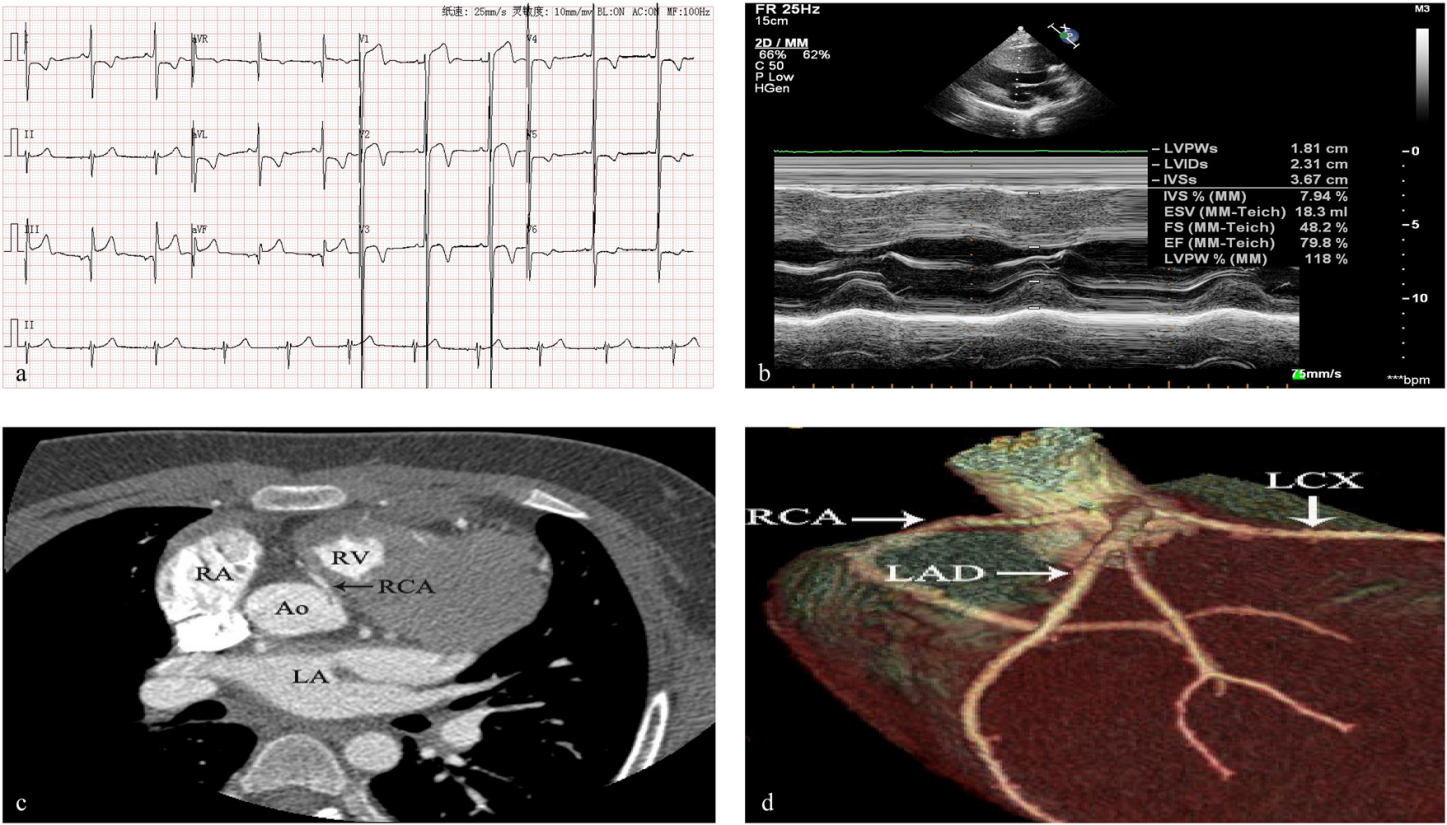
a EKG revealed sinus rate, prolonged QT duration time, abnormal Q wave, and flatness of ST segment and T wave. b Echocardiography showed interventricular septum and left ventricular posterior wall hypertrophy. c and d Computed tomography coronary angiography revealed RCA originated from left coronary sinus.

A 12-year-old male was admitted to our hospital because of intermittent chest pain for 1 year. There was no sudden cardiac death, HCM or coronary artery disease in his parents. However, his granduncle had HCM history and died at the age of 67. On physical assessment, his respiratory rate was 20 per minute, heart rate was 75 per minute, blood pressure was 120/62 mmHg and SpO_2_ was 99 %. Physical examination of the heart and lungs was unremarkable. Laboratory tests yielded a normal complete blood count, basic metabolic profile, normal troponin levels, and NT-proBNP. The tandem mass spectrum of blood and urine was normal. An ECG revealed sinus rate, prolonged QT duration time (QT interval=458 ms), abnormal Q wave and flatness of ST segment and T wave (Figure 2a). The echocardiogram revealed left ventricular hypertrophy, interventricular septal thickness was 3.40 cm (z-score=24.98) in diastole and 3.67 cm (z-score=15.5) in systole, left ventricular free wall thickness was 1.00 cm (z-score=2.21) in diastole and 1.81 cm (z-score=3.31) in systole, and the EF was normal (EF=79.8 %) (Figure 2b). Coronary CTA revealed right coronary artery (RCA) originated from left sinus (Figure 2c and d). The patient was treated with β-blockers and suggested to avoid physical activity. He has no symptoms after 2 years follow-up.

## Ethics approval

This study involving the collection and analysis of patient case data was conducted in accordance with ethical principles, and all procedures were carried out following the Declaration of Helsinki. The research protocol and data collection methods were assessed, and it was determined that formal ethics approval was not needed for this case report. The study strictly adhered to patient confidentiality and privacy standards. Written consent was obtained from the parents (also the legal guardians) of the child patient, and all the data were anonymized to ensure the protection of personal information.

## Consent for publication

Written informed consent for publication of their clinical details and clinical images was obtained from the patient’s parents of the patient.

## Genetic testing

**Figure 3:**
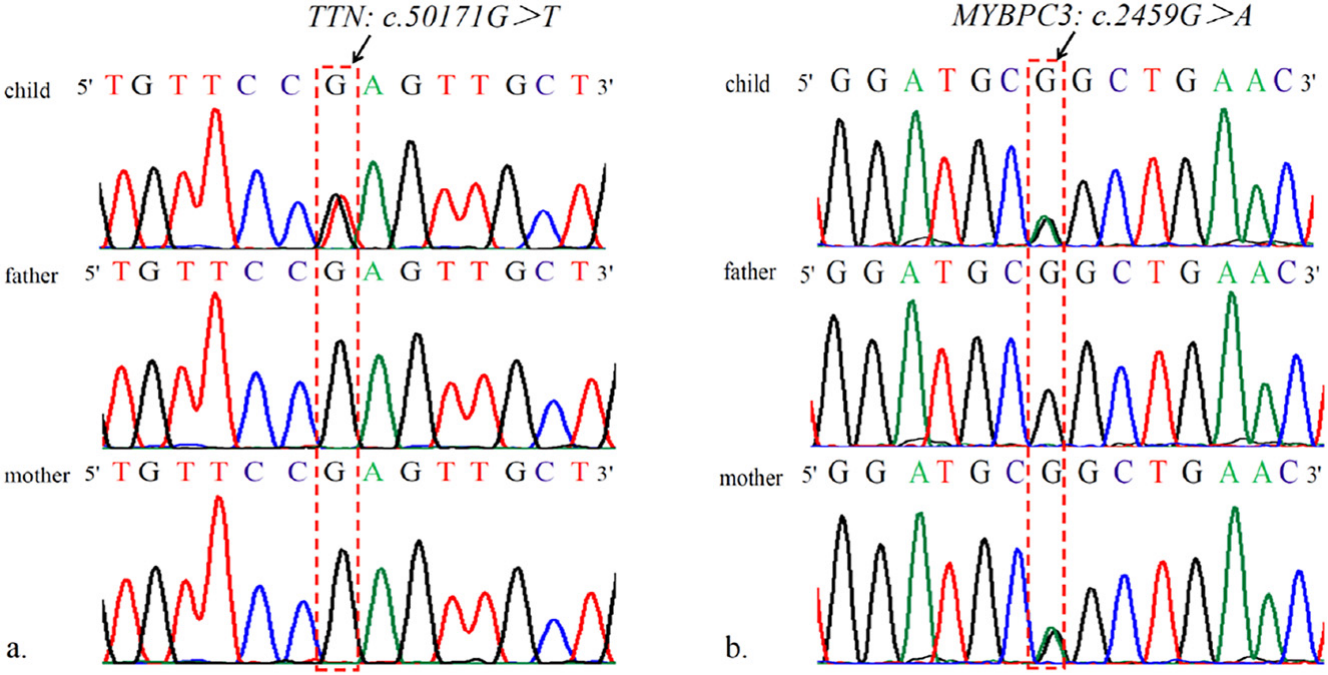
Sanger sequence analysis. a *c.50171G>T* heterozygous mutation of *TTN* gene in Case 1. b *c.2459G>A* heterozygous mutation of *MYBPC3* gene in Case 2 and the patient’s mother.

Construction of DNA Libraries and Sequencing Exome sequencing was performed according to the manufacturer’s instructions. Briefly, Genomic DNA was extracted from peripheral venous blood samples using whole-blood genomic DNA extraction kit (Tiangen, China). For whole exome sequencing 1 µg DNA was used for library preparation using the TruSeq DNA LT Sample Prep Kit v2 according to the manufacturer’s instructions followed by hybridization using Nimblegen SeqCap EZ Exome v3 (Roche) and Paired-end Sequencing (2 × 100 bp) on the illumina HiSeq 2500 with TruSeq v3 chemistry. A *c.50171G>T* (*p.R16724L*) heterozygous mutation of Titin gene (*TTN*) is found by WES and confirmed with Sanger sequencing in Case 1 (Figure 3a). A *c.2459G>A* (*p.R820Q*) heterozygous mutation of myosin-binding protein C gene (*MYBPC3*) is confirmed in Case 2 and his mother (Figure 3b).

## Discussion

Coronary artery development is a delicate, complex, and finely tuned process and the origin and distribution of coronary artery is developed in embryonic 4–6 weeks [12]. Coronary angiography was performed in 110,158 patients with coronary artery disease, and anomalous origin of coronary artery was found in 835 cases [2]. RCA originated from left coronary artery (LCA) sinus was the most common subtype (39.28 %, 328 in 835 cases) [2]. Coronary angiography was also performed in 515 children with congenital heart disease, and abnormal origin of coronary artery was found in 42 cases [13]. RCA originated from LCA sinus accounted for 38.1 % [13]. Although CAA is a very rare, RCA origin anomalies is most common in CAA. RCA that arises from left anterior sinus, with anomalous course, which includes (1) Posterior atrioventricular groove or retrocardiac, (2) Retroaortic, (3) Between aorta and pulmonary artery (intramural), (4) Intraseptal, (5) Anterior to pulmonary outflow and (6) Posteroanterior interventricular groove (wraparound) [14]. The angle between the RCA opening and aortic wall in RCA arising from contralateral coronary sinus is always acute angle. The mechanism of sudden cardiac death is caused by RCA occlusion due to aortic dilation during exercise [14]. Patients with an anomalous coronary artery originating from the opposite sinus of valsalva taking an inter-arterial course have higher rates of both myocardial infarction and surgical revascularisation during long-term follow up [15]. RCA was originated from the left coronary sinus and distributed intramural aortic in case 1 by echocardiography and coronary CTA, and the degree of lumen stenosis is greater than 50 %. RCA was originated from left coronary sinus with inter-arterial course, but no sign of lumen stenosis in case 2.

A total of 7 cases with RCA originated from left coronary sinus coexisted with HCM, have been reported with our literature review, and only one of them was detected DNA sequencing (see Table 1) [5], [6], [7], [8], [9], [10], [11]. Case 1 is the youngest HCM case accompanied with CAA until now. Interestingly, the patient presented the phenotype similar with restrictive cardiomyopathy (RCM). The echocardiogram of Case 1 revealed the dilated double atria and comparatively small ventricles. HCM exhibited RCM phenotype is very rarely [16], [17], [18], [19], [20], the 2 forms may represent different phenotypes of the same genetic disease [21]. And the gene mutation site of *TTN* of the patients has never been reported in previous HCM cases. HCM is a hereditary disease characterized by cardiac hypertrophy with diastolic dysfunction. HCM develops into the stage of severe heart failure, and some patients may present abnormal changes in the morphology and function of RCM. Gene mutations causing HCM have been found in about half of HCM patients, while the genetic etiology and pathogenesis remain unknown for many cases of HCM [22]. Of the patients with positive genetic testing, most disease-causing variants occur in myosin heavy chain and MYBPC3 [23], [24], [25]. Recently, study revealed that the mutations found in familial HCM increased binding of titin to muscle-specific ring finger protein 1 (MURF1) and enhanced titin degradation by ubiquitination [22]. Titin-Truncating variants increase the risk of cardiovascular death in patients with HCM [26]. Few literatures have reported that hypertrophic cardiomyopathy presents restrictive cardiomyopathy changes which may be due to some genetic and/or environmental factors and the genetic/phenotypic heterogeneity of HCM [20]. The “restrictive phenotype” is an uncommon presentation of the clinical spectrum of HCM and is associated with severe limitation and poor prognosis [16].

**Table 1:** Current reported cases of RCA originated from left coronary sinus and HCM.

Reference	Sex, age	Manifestation	Type of HCM	Obstruction	Genetic testing	Treatment
Zheng et al. [5]	Male, 26y	Atypical chest pain	Interventricular septum. Combined with VSD	No	–	Angiotensin-converting enzyme inhibitors and Beta blockers
Moza et al. [6]	Male, 29y	Anginal chest pain 、recurrent syncopal attacks	Asymmetric septal	Yes	–	CABG. Subaortic septal myomectomy and resection of an anomalous papillary muscle
Tyczyński et al. [7]	-, 65y	–	Asymmetric septal	Yes	–	Alcohol septal ablation, no surgical correction was done to the anomalous RCA
Afari et al. [8]	Male, 18y	Collapsed while playing basketball (SCA)	Apical	No	MT-TK gene mutation	ICD. A surgical unroofing of the anomalous RCA 5 years later after the SCA, and medical thetapy with β-blocker
Liddy et al. [9]	Male, 40y	Atypical chest pain of 1-month duration	Asymmetric septal	Yes	–	Medical management with bisoprolol
Yalçin et al. [10]	Male, 20y	Prolonged chest pain and shortness of breath	Asymmetric septal	No	–	β-blocker therapy
Dermengiu et al. [11]	Male, 20y	Collapsed during soccer practice (SCD)	Asymmetric septal	Unknown	–	Death

VSD, ventricular septal defect; CABG, coronary artery bypass grafting; HCM, hypertrophic cardiomyopathy; ICD, implantable cardioverter defibrillator; RCA, right coronary artery; SCA, sudden cardiac arrest; SCD, sudden cardiac death.
